# Kidscreen-27 in assessment quality of life adolescents with idiopathic scoliosis

**DOI:** 10.1186/1748-7161-9-S1-O73

**Published:** 2014-12-04

**Authors:** Edyta Kinel, Anna Podolska-Piechocka, Tomasz Kotwicki, Przemyslaw Lisinski

**Affiliations:** 1 Department of Rheumatology and Rehabilitation, Clinic of Rehabilitation, University of Medical Sciences, Poznan, Poland; 2 Department of Paediatric Orthopaedics and Traumatology, University of Medical Sciences, Poznan, Poland

## Background

There are a few quality of life (QoL) questionnaires dedicated for patients with adolescent idiopathic scoliosis (AIS): Brace Questionnaire (BrQ), SRS-22, Scoliosis Quality of Life Index (SQLI) and SF-36. Kidscreen-27 generic health related QoL life measures for children and adolescents.

## Aim

The aim was to evaluate the quality of life of adolescents with idiopathic scoliosis in comparison with corresponding healthy adolescents. In each case the responses were also gained from the parents/ care-givers.

## Design

Cross-sectional study. It involved 82 adolescents, ages ranging between 11.0 and 16.0 years, all with IS with Cobb angle between 20-45 degrees. Adolescents were wearing the Chêneau orthosis, (more than 3 months for at least 12h per day). The control group consisted of 82 healthy adolescents, (11.0-16.0 years) and their parents/care-givers.

## Methods

Kidscreen-27 consists of five Rasch scaled dimensions: Physical Well-Being (5 items), Psychological Well-Being (7 items), Autonomy & Parents (7 items), Peers & Social Support (4 items), and School Environment (4by items). Answers are pointed from 0 to 4. The higher the score the better the QoL. In the evaluation Kidscreen-27 questionnaire was used for AIS and healthy adolescents. Kidscreen-27 for parents/ care-givers was used additionally. Answering the Kidscreen-27 require 10-15 minutes.

## Results

The age of examined group with AIS was 13.5 ± 1.6 years. Cobb angle was 31.0 ± 8.1 degrees. The age of control group was 13.6 ± 1.7 years. In dimension, Peers & Social Support, AIS and their parents/care-givers achieved lower results (10.65 ± 3.13 AIS and 9.13± 3.09 parents/care-givers) than in other dimensions. There were significant differences between AIS and their parents/care-givers in dimensions of Physical Well-Being (p=0.023) and Peers & Social Support (p<0.001). Analyze of Autonomy & Parents dimension showed significant difference between AIS and control group and their parents/care-givers (p=0.032 AIS and p=0.014 control group).

## Conclusions

Patients with AIS showed better autonomy and relations with parents/care-givers. Parents/care-givers of patients with AIS also presented good autonomy and relations with children.

## References

[B1] Mazur J Małkowska-Szkutnik A Dzielska A Tabak I Polska wersja kwestionariuszy do badania jakości życia związanej ze zdrowiem dzieci i młodzieży (KIDSCREEN) 2008 Instytut Matki i Dziecka, Warszawa

[B2] The Kidscreen Group Europe The Kidscreen Questionnaires Quality of life questionnaires for children and adolescents 2006 Pabst Science Publishers, Lengerich, Germany

